# Laquinimod Inhibits Inflammation-Induced Angiogenesis in the Cornea

**DOI:** 10.3389/fmed.2020.598056

**Published:** 2020-11-10

**Authors:** Zuohong Li, Jianping Chen, Lei Lei, Nan Jiang, Yanling Zhu, Yu Jia, Yehong Zhuo, Wenru Su

**Affiliations:** ^1^State Key Laboratory of Ophthalmology, Zhongshan Ophthalmic Center, Sun Yat-sen University, Guangzhou, China; ^2^Department of Pediatric Ophthalmology, Guangzhou Children's Hospital and Guangzhou Women and Children's Medical Center, Guangzhou Medical University, Guangzhou, China

**Keywords:** corneal neovascularization, cytokines, inflammation-induced angiogenesis, macrophage, laquinimod

## Abstract

**Background:** Inflammation-induced angiogenesis plays a critical role in many eye diseases, and abnormal angiogenesis inhibition is regarded as a therapeutic approach. Here, we examined the effects of laquinimod on inflammatory corneal angiogenesis.

**Methods:** Mouse model of corneal neovascularization was induced by NaOH. Laquinimod or control vehicle were topically applied to alkali-treated eyes twice a day for 10 days. Corneal neovascularization, infiltrating inflammatory cells, and the levels of chemokines, pro-inflammatory cytokines were assessed. RAW cells and human umbilical vein endothelial cells were used *in vitro* to further explore the underlying mechanisms of the effects of laquinimod on inflammation-induced angiogenesis.

**Results:** Topical administration of laquinimod to the injured corneas dramatically inhibited alkali-induced corneal neovascularization and decreased inflammatory cell (such as macrophage) infiltration in a corneal injury mouse model. Laquinimod significantly downregulated the expression of chemokines (monocyte chemotactic protein-1 and macrophage inflammatory protein-1), pro-inflammatory cytokines (interleukin-1β and tumor necrosis factor-alpha), vascular endothelial growth factor, nucleotide-binding oligomerization domain-like receptor family pyrin domain-containing 3 and apoptosis-associated speck-like protein containing C-terminal caspase-recruitment domain adaptor protein in both injured corneas and RAW cells. *In vitro*, laquinimod also dramatically inhibited the proliferation, migration and tube formation of human umbilical vein endothelial cells.

**Conclusion:** Laquinimod inhibits inflammation-induced angiogenesis in the cornea. These results suggest that laquinimod is a potential new therapeutic option for corneal neovascularization and other angiogenesis-associated diseases.

## Introduction

Angiogenesis is vital for normal physiological processes and tissue repair, including growth, reproduction and wound healing (1, 2). However, the avascular status and corneal clarity are crucial for visual acuity (3). Angiogenesis and the accompanying inflammatory responses induce disorganization of collagen fibrils and corneal neovascularization (CNV) and impair corneal clarity and vision (4). Furthermore, inflammatory mediators induce keratocytes to produce extracellular matrix in the corneal stroma, causing permanent vision loss (4). Thus, the development of new strategies that inhibit inflammation-induced angiogenesis has become the primary goal for treating many corneal diseases.

The inflammatory response involves complicated cell interactions and chemotactic signals, which are regulated by a balance of pro-inflammatory and anti-inflammatory factors (5). During the inflammatory response, monocytes are recruited into inflamed tissue and differentiate into macrophages (5). Macrophages and other inflammatory cell types secrete various proangiogenic and pro-inflammatory factors and markedly promote the inflammatory response by interacting with vascular tip cells, endothelial cells, progenitor cells and epithelial cells (5–8). Furthermore, some studies have suggested that macrophages play a pivotal role in the process of inflammatory CNV by providing proangiogenic and pro-inflammatory cytokines (9, 10). Inhibiting macrophage infiltration largely suppresses the development of CNV (11). For that reason, understanding the functions and regulatory mechanisms of macrophages is important for better therapy for inflammatory neovascularization.

Laquinimod, a small molecule with a molecular weight of 357 Da, was licensed as immunomodulatory from Teva (Petach Tikva, Israel) in 2004 (12). Laquinimod is an oral immunomodulatory drug that is under development for the treatment of multiple sclerosis (MS), lupus nephritis, Crohn's disease, and Huntington's disease (13–18). Further studies indicated that laquinimod suppresses the infiltration of monocytes in experimental autoimmune encephalomyelitis (EAE) by inhibiting chemokine signals, such as CC chemokine receptor 2 (CCR2) and CC chemokine ligand 2 (CCL2), for monocyte chemotaxis (19). In addition, laquinimod inhibits phosphorylation of the p38/MAPK and JNK signaling pathways in human monocytes *in vitro* (19). Since laquinimod inhibits infiltrating monocyte functions to modulate inflammatory processes by regulating various chemotactic and pro-inflammatory factors, we investigated the effect of laquinimod on macrophages and a mouse model of inflammation-induced CNV.

## Materials and Methods

### Ethics Statement and Animals

Female C57BL/6 mice aged 6 to 8 weeks were used for the corneal injury model, and the mice were obtained from the Animal Laboratory of Zhongshan Ophthalmic Center (Guangzhou, China) and housed in a specific pathogen-free facility. Animal care and use were in compliance with the Association for Research in Vision and Ophthalmology's Statement for the Use of Animals in Ophthalmic and Vision Research. All animal experiments were carried out according to the guidelines of the Animal Ethical Committee at Zhongshan Ophthalmic Center of Sun Yat-sen University (Permit Number 2017-094). All experimental animals were kept in a humidity- and temperature-controlled room and given plenty of food and water. Animal care staff and veterinary personnel monitored the health of all animals daily and kept them warm at all times. All efforts were made to minimize suffering.

### Corneal Neovascularization Induction and Treatment

C57BL/6 mice were intraperitoneally anesthetized with 4.3% kessodrate and received topically proparacaine (Alcaine, Alcon-Couvreur, Rijksweg, Belgium) for corneal analgesia. The right eye of each mouse was exposed to a 2-mm disk of filter paper with 0.5 N NaOH for 40 s. Then, the cornea was extensively rinsed by 25 mL of sterile saline. The corneal epithelia were gently scraped in a rotary motion parallel to the corneal limbus using a corneal knife without injuring the underlying corneal stroma. Five microliters of laquinimod (0.01, 0.05, or 0.25 mg/mL in endotoxin-free PBS) was topically applied to alkali-treated eyes twice a day for 14 days, and PBS was utilized as a control. The corneas were removed at day 3 after alkali burn, and the total RNA was extracted from the corneas by using the RNA Purification Kit (Yishan Biotechnology, Shanghai, China).

### CNV Assessment

On the 7 and 14th days after alkali injury, pictures of the corneas were taken by using a digital camera (ORCA ER; Hamamatsu, Japan), which was connected to the slit lamp (Zeiss, Jena, Germany). The CNV area was quantified using the following equation: area (mm^2^) = C/12 × 3.1416 × [R^2^-(R-L)^2^] (20), where C is the clock hour of CNV, R is the radius of the mouse cornea (*R* = 1.5 mm) and L is the average vessel length sprouting from the limbal vasculature. The percentage of CNV was the CNV area of each eye compared to the area of the entire cornea.

### Assessment of Neovascularization in Flat-Mounted Corneas

Female C57BL/6 mice were used for the corneal injury model and topically applied with PBS or laquinimod (0.25 mg/mL). The eyes were harvested on the 7 or 14th day after alkali injury and fixed in 4% paraformaldehyde overnight at 4°C. Corneas were dissected and incised by four incisions after the rinse in PBS. Corneas were blocked with 3% BSA in PBS with 0.3% Triton X-100 2 h at room temperature and incubated overnight at 4°C with a rabbit anti-CD31 antibody (Abcam, ab28364, 1:100). After three rinses in PBS with 0.3% Triton X-100, the detection of bound anti-CD31 antibodies was performed using anti-rabbit IgG Alexa Fluor 488 (Cell Signaling Technology, Germany, 1:1000) for 2 h at room temperature. Corneas were flat mounted on glass slides after three rinses in PBS and mounted with antifade mounting medium (Beyotime, Shanghai, China). Images of the flat mounts were captured with fluorescence microscopy (DMI3000 B; Leica, Wetzlar, Germany).

The innermost vessel of the limbal arcade was outlined to calculate the total area of the cornea in a masked fashion using ImageJ software. The vascular area was calculated as the difference between the avascular area and the total corneal area. And we used the ratio between the vascular area and total corneal area as statistical analysis of CNV to avoid errors caused by different size of cornea.

### Evaluation of Infiltrating Inflammatory Cells

The eyes were harvested on the 7th day after alkali injury and fixed in 10% buffered formalin. Then, samples were embedded in paraffin after the dehydration with ethanol gradient. The slices (5 μm) of paraffin-embedded tissues were routinely stained with H&E to detect inflammatory cells. The numbers of inflammatory cells in the central region of each burned cornea were counted in five randomly selected fields of each stained section at 200× magnification by two masked observers separately.

### Immunofluorescent Analysis

The eyes were harvested on the 7th day after alkali injury and embedded in optimal cutting temperature (OCT) compound after the fixation with 4% paraformaldehyde overnight at 4°C. We blocked 5 μm OCT frozen sections with 3% BSA for 1 h at room temperature and incubated the sections overnight at 4°C with a rat anti-F4/80 antibody (Abcam, ab66440, 1:200), rat anti-CD11b antibody (Abcam, ab8878, 1:200) or rabbit anti-CD31 antibody (Abcam, ab28364, 1:100). Detection of bound anti-F4/80, anti-CD11b or anti-CD31 antibodies was performed using anti-rat IgG Alexa Fluor 488 (Cell Signaling Technology, Germany, 1:1000), anti-rat IgG Alexa Fluor 555 (Cell Signaling Technology, Germany, 1:1000) or anti-rabbit IgG Alexa Fluor 555 (Cell Signaling Technology, Germany, 1:1000). After counterstaining with DAPI (Abcam, ab228549), the sections were mounted with antifade mounting medium (Beyotime, Shanghai, China). The sections were viewed and photographed with fluorescence microscopy (DMI3000 B; Leica, Wetzlar, Germany).

### Trypan Blue

Trypan blue is negatively charged and can pass through only damaged membranes and be absorbed by only dead cells (21). The cytotoxic activity of laquinimod was examined by the trypan blue exclusion method on the RAW264.7 mouse macrophage cell line (Zhong Qiao Xin Zhou Biotechnology, Shanghai, China). RAW cells were cultured at a density of 1 × 10^6^ cells/well in a 6-well plate in growth medium and treated with laquinimod (0, 0.1, 1, 10, and 100 μM) in the presence of lipopolysaccharide (LPS) (100 ng/mL) for 24 h. Trypan blue dye (4%) was added to each well and incubated for 10 min at 37°C. Thirty random fields with at least 20 cells were observed at 200× magnification in each field.

### Enzyme-Linked Immunosorbent Assay (ELISA)

RAW264.7 cells were cultured with or without laquinimod (4, 20, and 100 μM) and 100 ng/mL LPS for 24 h. The supernatants were collected, and the levels of tumor necrosis factor-alpha (TNF-α) (Invitrogen; Cat# 88-7324-88) and interleukin-1β (IL-1β) (Invitrogen; Cat# 88-7013-88) were examined using ELISA kits according to the manufacturer's instructions.

### Cell-Counting Kit-8 (CCK-8) Assay

Human umbilical vein endothelial cells (HUVECs) were obtained from ScienceCell (Carlsbad, CA). Triplicate 0.2-mL cell suspensions were cultured at 1 × 10^4^ cells/well in round-bottom 96-well microtiter plates and treated with laquinimod (0, 4, 20, and 100 μM) at 37°C in a CO_2_ incubator. At 48 h, 10 μL of CCK-8 reagent (Dojindo, Japan) was added to each well, and the cells were further incubated for 1–2 h at 37°C. The absorbance at 450 nm in each well was determined using a microplate reader (Pharmacia, Sweden).

### Migration Assay

HUVECs were cultured in a 6-well plate at a density of 5 × 10^5^ cells/well in growth medium. After the cells reached 90% confluence in each well, scratches were made using a sterile tip. The monolayer of HUVECs was incubated with a migration assay buffer consisting of serum-free medium and laquinimod (0, 4, 20, and 100 μM). Images were captured at 0, 6, 12, and 24 h. The wound healing area was analyzed using ImageJ software.

### Tube Formation Assay

A tube formation assay was performed as previously described (22, 23). In brief, 50 mL Matrigel was added to each well in 96-well plates for 30 min at 37°C. HUVECs were seeded on Matrigel at 1 × 10^4^ cells/well with laquinimod (0, 4, 20, and 100 μM) in depleted medium for 12 h at 37°C in a 5% CO_2_ incubator. Images were taken with a microscope with a 10× objective. Tube formation was quantified using Angiogenesis Analyzer for ImageJ (24). Four fields were analyzed in each experiment and repeated three times. The number (Nb) of nodes, Nb of branches, Nb of segments, Nb of junctions, total (Tot) length, Tot segment length, and Tot branching length were used to analyze the quantification of tube formation.

### Real-Time Quantitative RT-PCR

RAW cells were cultured at a density of 5 × 10^5^ cells/well in a 6-well plate with growth medium and treated with laquinimod (0, 4, 20, and 100 μM) in the presence of LPS (100 ng/mL) for 8 h. Total RNA was extracted by using the RNA Purification Kit (Yishan Biotechnology, Shanghai, China) for further experiments.

Total RNA from corneas or cultured RAW cells was reverse-transcribed into cDNA. ChamQ SYBR Color qPCR Master Mix (Vazyme Biotechnology) was used to examine the mRNA level of testing molecules by real-time PCR. Then, the relative changes in mRNA expression were examined using 2^−ΔΔCt^ method. Primers (Table 1) were synthesized by Invitrogen Biotechnology Co., Ltd. (Shanghai, China). The mRNA level in each sample was calculated by normalization to the expression of mouse GAPDH.

**Table 1:** Primer sequences of mouse genes for real-time RT-PCR.

**Gene**	**Primer sequence (5′ to 3′)**	
MCP-1	Sense	TTAAAAACCTGGATCGGAACCAA
	Antisense	GCATTAGCTTCAGATTTACGGGT
MIP-1	Sense	TTCTCTGTACCATGACACTCTGC
	Antisense	CGTGGAATCTTCCGGCTGTAG
NLRP3	Sense	ATCAACAGGCGAGACCTCTG
	Antisense	GTCCTCCTGGCATACCATAGA
ASC	Sense	GACAGTGCAACTGCGAGAAG
	Antisense	CGACTCCAGATAGTAGCTGACAA
IL-1β	Sense	TTCAGGCAGGCAGTATCACTC
	Antisense	GAAGGTCCACGGGAAAGACAC
TNF-α	Sense	CAGGCGGTGCCTATGTCTC
	Antisense	CGATCACCCCGAAGTTCAGTAG
VEGF-A	Sense	GCACATAGAGAGAATGAGCTTCC
	Antisense	CTCCGCTCTGAACAAGGCT
GAPDH	Sense	TGACCTCAACTACATGGTCTACA
	Antisense	CTTCCCATTCTCGGCCTTG

### Statistical Analysis

All experiments with six mice in each group were repeated at least three times, and representative data are shown. The CNV area, wound healing and tube formation of HUVECs were analyzed using ImageJ software. Statistical analysis was performed with the GraphPad Prism 7.0 software (GraphPad Software Inc. San Diego, CA, USA). The quantitative data are presented as the mean ± S.E.M on all parameters determined in the study. The normality test was assessed for all data using Shapiro-Wilk normality test. Data were analyzed statistically using one- way ANOVA or 2-tailed Student's *t*-test. A value of *p* < 0.05 was accepted as statistically significant.

## Results

### Laquinimod Inhibited CNV in a Corneal Injury Mouse Model

**Figure 1:**
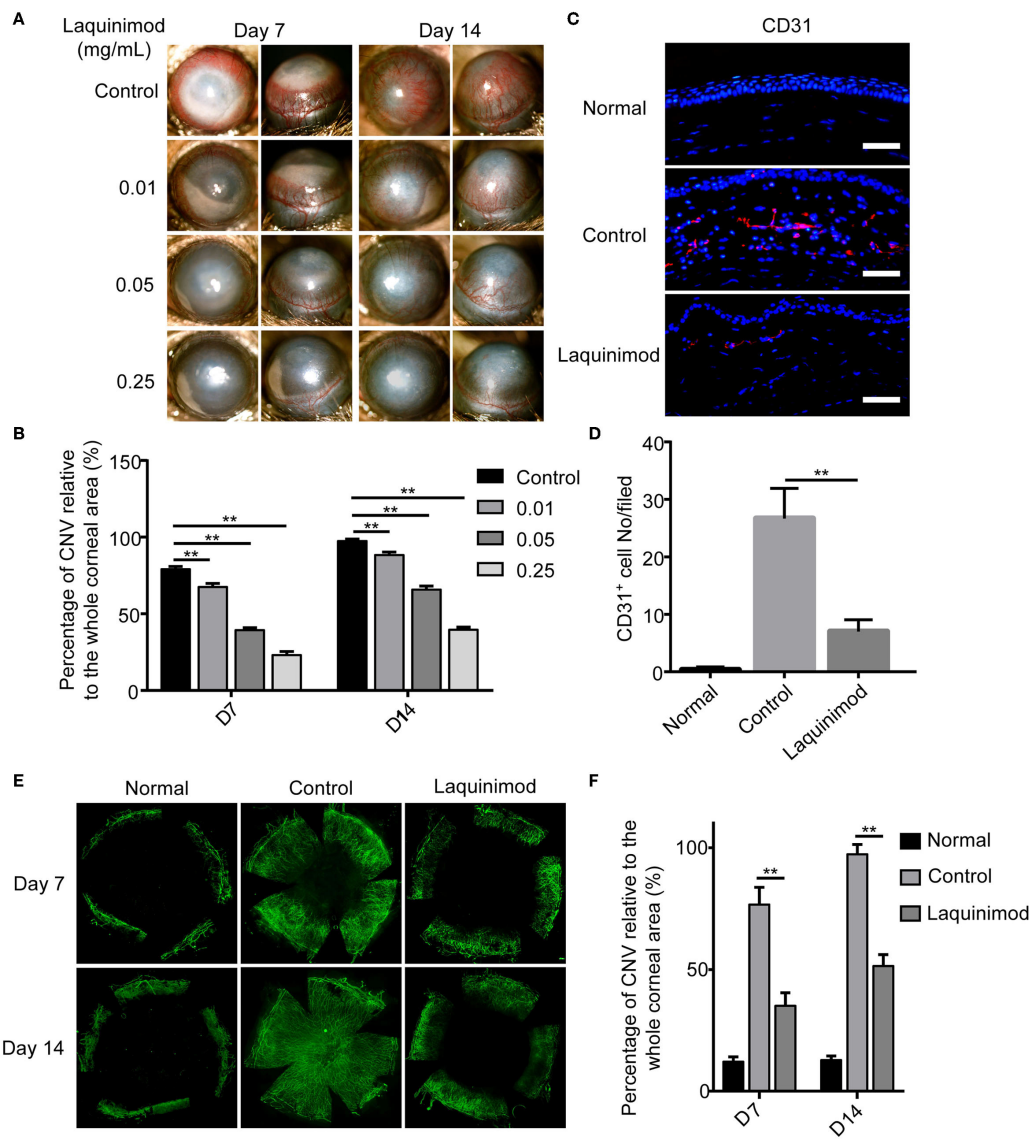
Topical laquinimod application attenuated CNV. **(A)** Five microliters of laquinimod (0.01, 0.05, and 0.25 mg/mL) was topically applied to alkali-injured corneas twice a day. Images were taken with a slit lamp to show the frontal (left panels) and lateral (right panels) views of each eye. **(B)** The area of CNV was normalized to the entire cornea on the 7 and 14th days after alkali injury. **(C)** Five microliters of PBS or laquinimod (0.25 mg/mL) was topically applied to alkali-treated eyes twice a day. The eyes were harvested on the 7th day after alkali injury. Corresponding cryosections from treated corneal tissues were stained with an anti-CD31 antibody. Original magnification: 200×. Scale bars = 50 μm. **(D)** Quantitative analysis of the data presented in **(C)**. **(E)** Five microliters of PBS or laquinimod (0.25 mg/mL) was topically applied to alkali-treated eyes twice a day for 14 days. Corneas were dissected on the 7 or 14th day after alkali injury and stained with anti-CD31 antibody. Images of the flat mounts were captured with fluorescence microscopy. **(F)** The total corneal area and avascular area were calculated in a masked fashion using ImageJ software. And the ratio between the vascular area and total corneal area was used to assess CNV in different groups. The data represent the means ± S.E.M.s (*n* = 6). PBS as control, **p < 0.01, laquinimod vs. control.

To examine the effect on angiogenesis, laquinimod (0.01, 0.05, or 0.25 mg/mL) was topically applied to alkali-injured corneas. Alkali burn resulted in significant CNV in the control group (Figure 1A). Nevertheless, laquinimod-treated eyes had less CNV than the control group and showed a dose-dependent suppression of CNV (Figure 1A). The area of CNV was normalized to the area of the entire cornea (21). The percentage of CNV in the laquinimod-treated eyes was significantly reduced compared to that in the control group (Figure 1B). Furthermore, immunofluorescent analysis of anti-CD31, a specific marker for endothelial cells, showed that CD31 staining in the control mice was increased compared to that in the normal mice and was significantly reduced in the laquinimod-treated group (0.25 mg/mL) 7 and 14 days after an alkali injury (Figures 1C–F). These data indicate that angiogenesis was significantly attenuated by treatment with laquinimod in alkali-injured corneas. During the experiment of alkali-induced mouse model, we observed that laquinimod (0.01, 0.05, or 0.25 mg/mL) had no significant toxicity to the corneal epithelium and did not affect corneal wound healing.

### Topical Laquinimod Application Inhibited Inflammatory Cells and F4/80- or CD11b-Positive Cells in Injured Corneas

**Figure 2:**
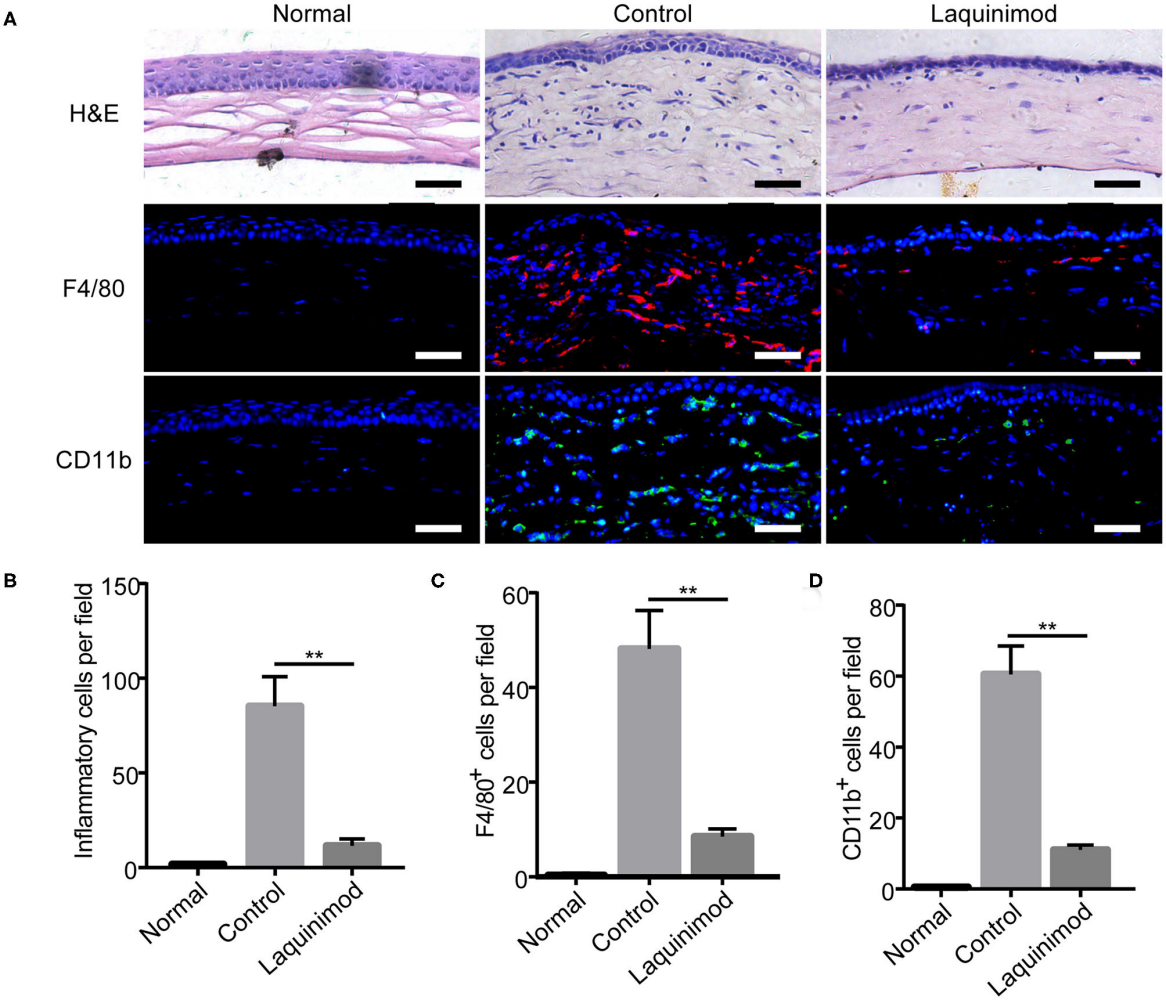
Topical laquinimod application inhibited inflammatory cells and F4/80- or CD11b-positive cells in injured corneas. Five microliters of laquinimod was topically applied to alkali-injured corneas twice a day. **(A)** The eyes were harvested on the 7th day after alkali injury. H&E-stained sections were obtained, and the number of inflammatory cells in the central region of each burned cornea was determined. Frozen OCT sections from treated corneal tissues were stained with an anti-F4/80 or anti-CD11b antibody. Scale bars = 50 μm. **(B–D)** Quantitative analysis of the data presented in **(A)**. (*n* = 6). PBS as control, **p < 0.01, laquinimod vs. control.

Macrophages and other inflammatory cell types play a pivotal role in the development of inflammatory CNV (9, 10, 25). To further understand the effect of laquinimod on CNV, we compared each group by H&E staining and immunofluorescent staining of F4/80 or CD11b in injured corneas. H&E staining showed that numerous inflammatory cells infiltrated the cornea after alkali injury but were noticeably decreased in the laquinimod-treated group (Figures 2A,B). Furthermore, the number of cells that were positive for F4/80 [a macrophage marker (26)] or CD11b [a molecule that controls monocyte migration (27)] was significantly increased in the alkali-injured cornea but was noticeably decreased in the laquinimod-treated group (Figures 2A,C,D). These data suggest that macrophages and other inflammatory cell types were significantly attenuated by laquinimod treatment in alkali-injured corneas.

### Inhibition of Chemotactic Factor, Inflammasome, and Pro-inflammatory Factor Expression by Laquinimod in Injured Corneas

**Figure 3:**
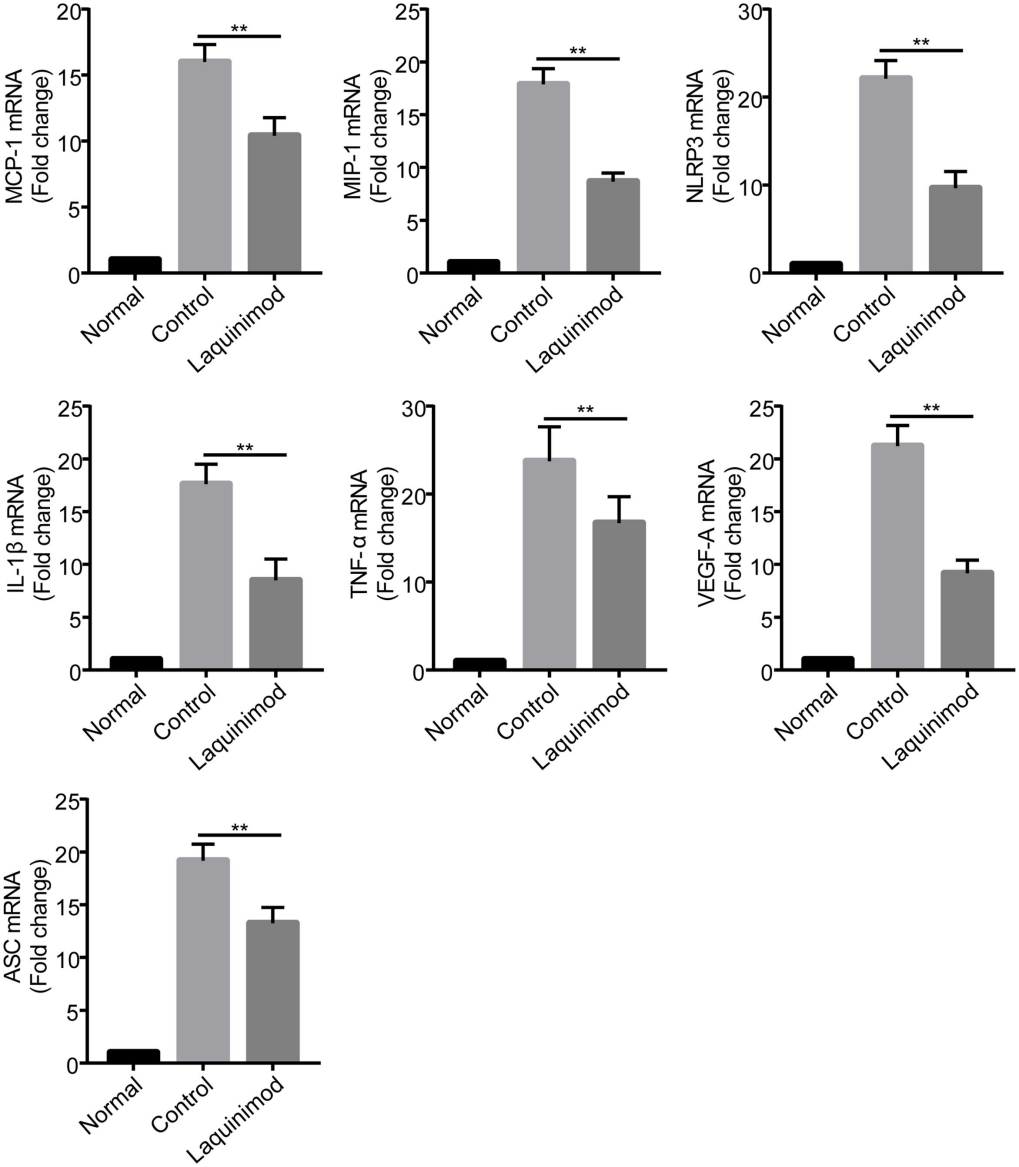
Inhibition of chemotactic factor, inflammasome, and pro-inflammatory factor expression by laquinimod in injured corneas. Five microliters of 0.25 mg/mL laquinimod or 5 μL of PBS was topically applied to alkali-injured corneas twice a day. The mRNA expression of chemokines (MCP-1 and MIP-1), pro-inflammatory cytokines (IL-1β and TNF-α), VEGF-A, NLRP3, and ASC in wound sites 3 days after injury was determined by quantitative RT-PCR. The results are expressed as the mean ± S.E.M. of the fold increase over the control (*n* = 5). PBS as control, **p < 0.01, laquinimod vs. control.

Inflammatory cells, such as macrophages, modulate neovascularization by releasing numerous chemotactic and pro-inflammatory cytokines (28). The previous results suggested that laquinimod inhibited inflammatory cells in injured corneas. Thus, to understand the mechanism of laquinimod in alkali-injured corneas, we next examined the effect of laquinimod on the expression of chemotactic and pro-inflammatory factors in alkali-injured corneas. The expression of chemokines [monocyte chemotactic protein-1 (MCP-1) and macrophage inflammatory protein-1 (MIP-1)], pro-inflammatory cytokines (IL-1β and TNF-α), vascular endothelial growth factor-A (VEGF-A), nucleotide-binding oligomerization domain-like receptor family pyrin domain-containing 3 (NLRP3) and apoptosis-associated speck-like protein containing C-terminal caspase-recruitment domain adaptor protein (ASC) were upregulated in alkali-injured corneas (Figure 3). However, the expression of chemotactic, inflammasome and pro-inflammatory factors was downregulated in the laquinimod-treated group. These data indicate that laquinimod attenuates angiogenesis by downregulating chemotactic, inflammasome and pro-inflammatory factors in alkali-injured corneas.

### Inhibition of Chemotactic Factor, Inflammasome, and Pro-inflammatory Factor Expression by Laquinimod in RAW Cells *in vitro*

**Figure 4:**
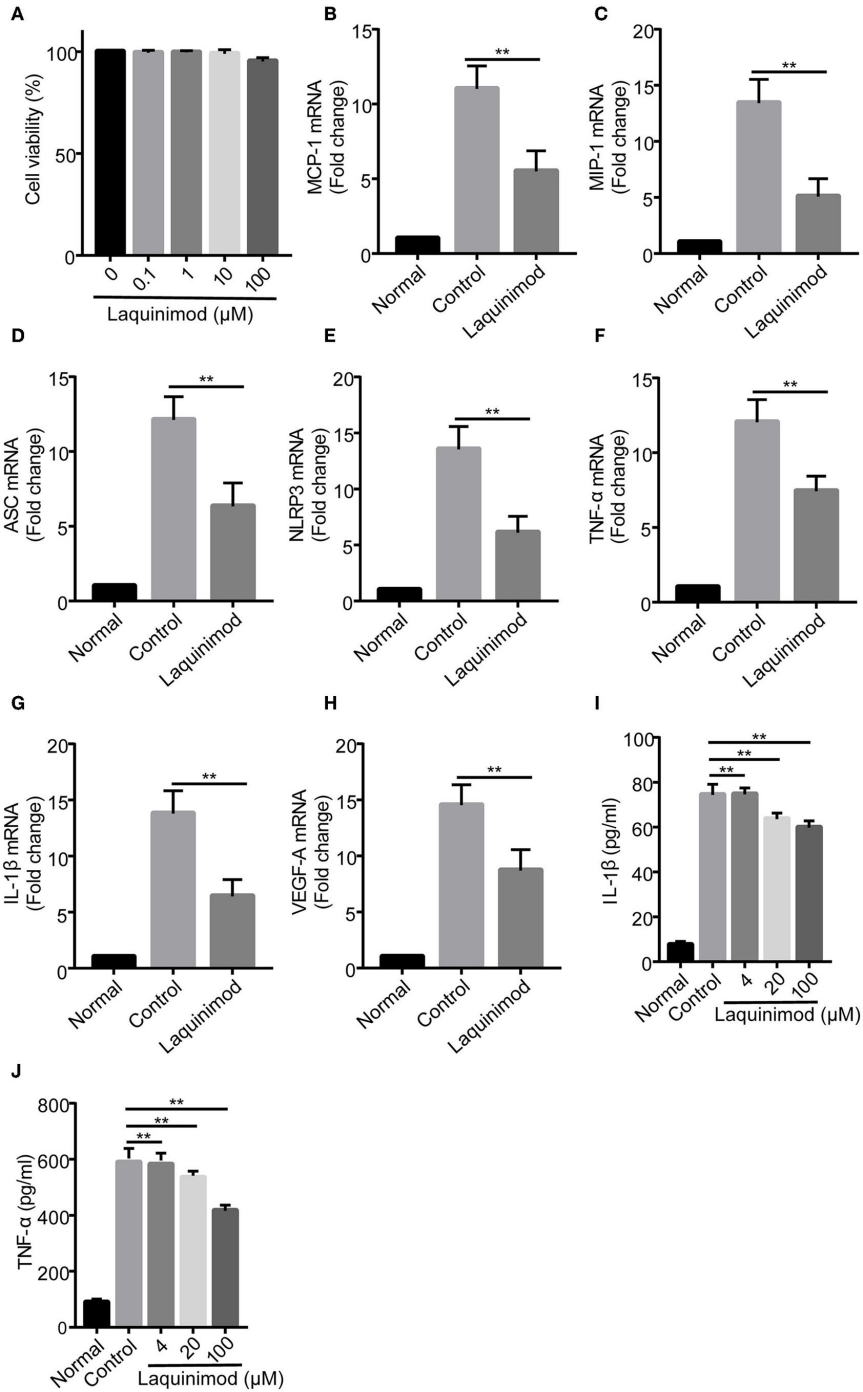
Chemotactic factor, inflammasome, and pro-inflammatory factor expression in laquinimod-treated RAW cells. **(A)** RAW cells were seeded in a 6-well plate at a density of 1 × 10^6^ cells/well in growth medium and treated with laquinimod (0, 0.1, 1, 10, and 100 μM) in the presence of LPS for 24 h. After treatment, the cytotoxic activity of laquinimod was evaluated by trypan blue dye exclusion assay. **(B–H)** RAW cells were seeded in a 6-well plate at a density of 5 × 10^5^ cells/well in growth medium and treated with laquinimod (0, 4, 20, and 100 μM) in the presence of LPS (100 ng/mL) for 8 h. The cell lysates and RNA were collected. The mRNA levels of chemokines (MCP-1 and MIP-1), pro-inflammatory cytokines (IL-1β and TNF-α), VEGF-A, NLRP3, and ASC were detected by RT-PCR and normalized to GAPDH. **(I,J)** RAW264.7 cells were cultured with or without laquinimod (4, 20, and 100 μM) and 100 ng/mL LPS for 24 h. The secretion of IL-1β and TNF-α in RAW 264.7 were examined by ELISA after the treatment of laquinimod. The results are expressed as the mean ± S.E.M. PBS as control, **p < 0.01, laquinimod vs. control.

To further explore whether laquinimod affects inflammatory neovascularization by regulating the functions of macrophages, we examined the effect of laquinimod on the expression of chemotactic and pro-inflammatory factors in the RAW264.7 mouse macrophage cell line. The trypan blue exclusion data showed that laquinimod did not affect the viability of RAW cells *in vitro* (Figure 4A). Similar to the expression of testing molecules in alkali burn wounds, laquinimod decreased the mRNA expression of LPS-induced chemokines (MCP-1 and MIP-1), pro-inflammatory cytokines (IL-1β and TNF-α), VEGF-A, NLRP3 and ASC in RAW cells *in vitro* (Figures 4B–H). Furthermore, secretion of the pro-inflammatory cytokines IL-1β and TNF-α were decreased in laquinimod-treated macrophages compared with the control group (Figures 4I,J). These data indicate that laquinimod attenuates angiogenesis by downregulating macrophage chemotactic, inflammasome and pro-inflammatory factors in alkali-injured corneas.

### The Effect of Laquinimod on HUVEC Proliferation and Migration

**Figure 5:**
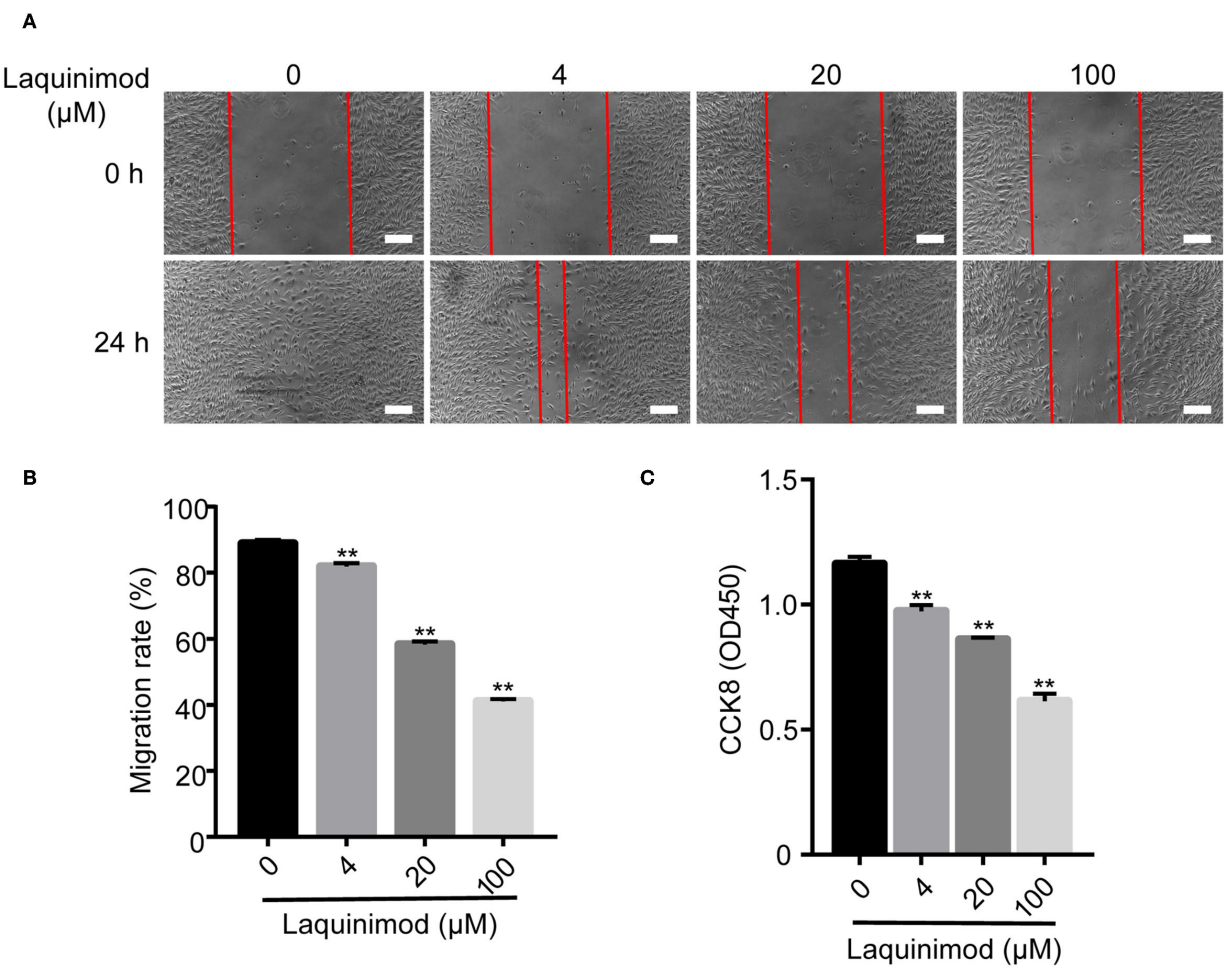
Inhibition of HUVEC proliferation and migration by laquinimod. **(A)** Images of HUVEC migration, which were assessed in a scratch assay, were captured at the same position at 0, 12, and 24 h after incubation with migration assay buffer containing laquinimod or PBS. Scale bars = 100 μm. **(B)** The area of the wound was calculated with ImageJ software. **(C)** HUVECs (1 × 10^5^) were cultured in the presence of laquinimod at 37°C in a CO_2_ incubator, and cell proliferation was determined by a CCK-8 assay. The results from one representative experiment of four are shown (in triplicate). PBS as control, **p < 0.01, laquinimod vs. PBS-treated cells.

The proliferation and motility of vascular endothelial cells are vital in angiogenesis (29). Thus, we examined the role of laquinimod in the proliferation and motility of HUVECs by CCK-8 and scratch assays. Cell proliferation was inhibited in laquinimod-treated cells compared to in control cells (Figure 5C). Furthermore, the healing area of HUVECs in the laquinimod-treated group was reduced compared with that in the control group at the 24 h time point in the scratch assays (Figures 5A,B).

### Laquinimod Blocked Tube Formation of HUVECs

**Figure 6:**
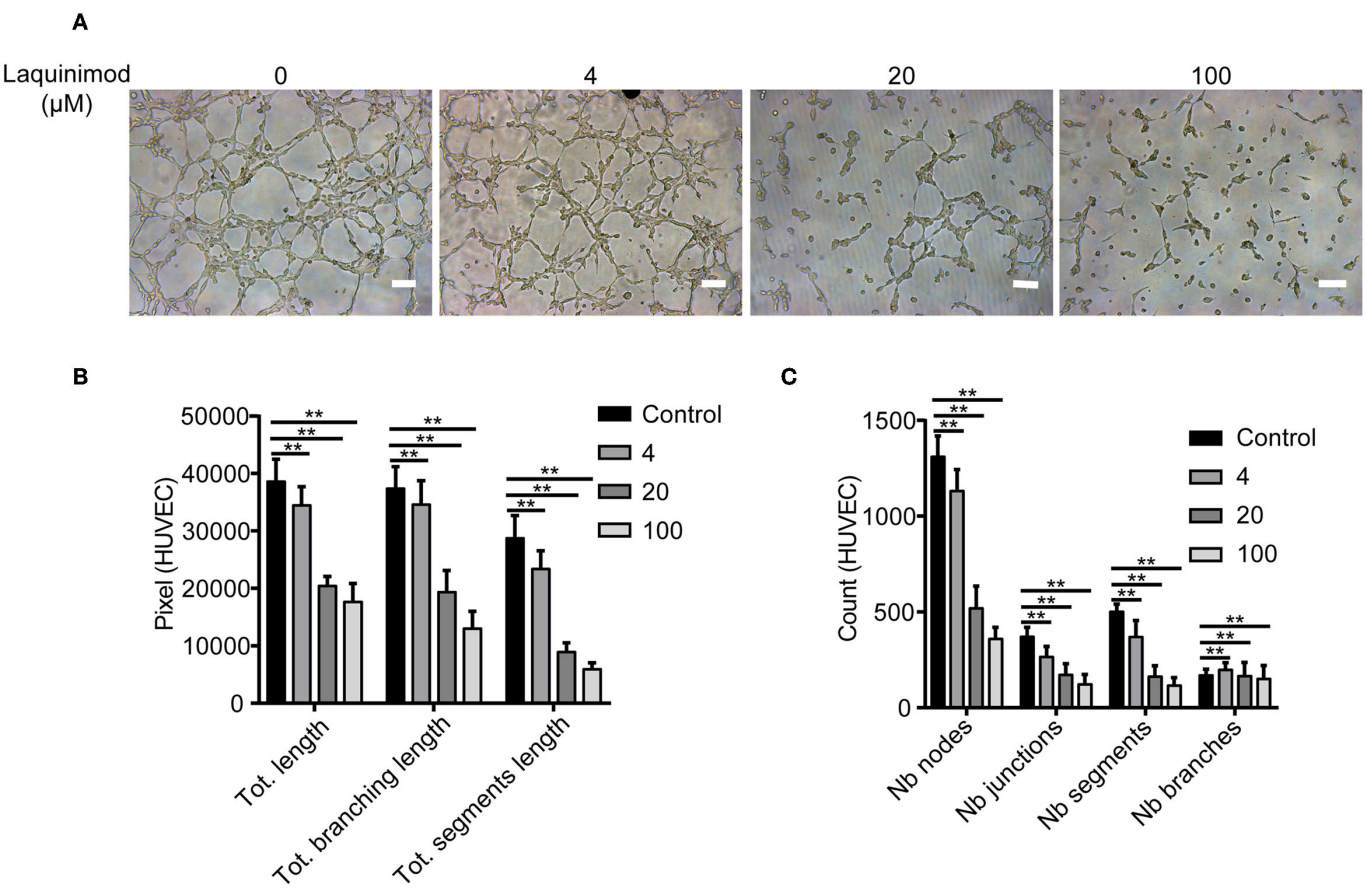
The effect of laquinimod on tube formation in HUVECs. **(A)** HUVECs (10,000 cells/well) were seeded on Matrigel containing laquinimod in depleted medium. The cells were cultured for 12 h at 37°C and 5% CO_2_. Photos were taken with an inverted microscope using a 10× objective (four images per group). Scale bars = 100 μm. **(B,C)** Tube formation was quantified by using Angiogenesis Analyzer for ImageJ. These data were analyzed, and all values were normalized to the analyzed area. The Nb of nodes, Nb of junctions, Nb of segments, Nb of branches, Tot length, Tot branching length, and Tot segment length were determined. PBS as control, **p < 0.01, laquinimod vs. PBS-treated cells.

Tube formation of blood endothelial cells plays a critical role in neovascularization (30). The effect of laquinimod on the tube formation of HUVECs was examined *in vitro*. We analyzed tube formation of HUVECs using Angiogenesis Analyzer for ImageJ. The Nb of nodes, Nb of branches, Nb of segments, Nb of junctions, Tot length, Tot segment length, and Tot branching length were used to analyze the quantification of tube formation. These results showed that laquinimod attenuates tube formation (Figure 6).

## Discussion

Abnormal angiogenesis causes vision impairment in ~1.4 million individuals in America each year (31, 32). Therefore, the regulation of angiogenesis is a valuable therapeutic strategy for many ocular diseases. Currently, steroids and other anti-inflammatory drugs are widely used, but these treatments are associated with unstable efficacies and significant side effects (33). Anti-VEGF biological agents have shown effective therapeutic effects in numerous ocular diseases (34–36). However, these biological agents are accompanied by severe complications in the eye, such as delaying epithelial wound healing and persistent epithelial defects or even corneal dissolution (2, 37, 38). Thus, complementary therapeutics for inhibiting angiogenesis are necessary. In this study, our results indicated that topical treatment with laquinimod significantly reduced alkali-induced CNV (Figure 1), suggesting that laquinimod has therapeutic potential in inflammation-induced angiogenesis.

Inflammation is closely linked to neovascularization, especially during tissue damage (39). Neovascularization plays a key role in the regeneration of injured tissue by transporting inflammatory cells and nutrients. Inflammatory cells further facilitate angiogenesis by pro-inflammatory cytokines and chemokines (1). Macrophages are inflammatory cells that interact with angiogenic effector cells to modulate neovascularization, especially in avascular tissues such as the cornea (11, 28, 40, 41). In this study, we observed that the number of infiltrating inflammatory cells in alkali-injured corneas was markedly reduced in the laquinimod-treated group (Figures 2A,B). Furthermore, topical application of laquinimod markedly reduced F4/80+ and CD11b+ cells in alkali-injured corneas (Figures 2A,C,D). These data suggest that laquinimod attenuates inflammatory CNV by inhibiting infiltrating inflammatory cells, such as macrophages.

Macrophages and other inflammatory cell types interact with angiogenic effector cells to modulate neovascularization by producing numerous chemotactic and pro-inflammatory cytokines (28). MCP-1 and MIP-1α, two well-studied CC chemokines, regulate the recruitment of monocytes and induce CNV (42). Moreover, NLRP3 and ASC are inflammasome sensors that induce the production of the pro-inflammatory cytokines IL-1β and TNF-α in monocytes (43, 44). The release of IL-1β, TNF-α, and VEGF by activated macrophages modifies the local extracellular matrix and promotes endothelial cell migration or proliferation in angiogenesis (45). In this study, topical treatment with laquinimod significantly downregulated the mRNA expression of chemokines (MCP-1 and MIP-1), pro-inflammatory cytokines (IL-1β and TNF-α), VEGF-A, NLRP3, and ASC in corneal wounds (Figure 3). Similar to the expression in alkali burn wounds, laquinimod decreased the mRNA expression of chemokines, inflammasomes and pro-inflammatory cytokines in RAW cells (Figure 4). These data suggest that laquinimod attenuates CNV by suppressing the expression of chemokines, inflammasomes and pro-inflammatory factors in macrophages.

CNV is caused by corneal infections, inflammation, trauma, chemical damage, and misuse of contact lenses (46–48). The neovascularization process contains multiple events, including proliferation, migration, sprouting, and tube formation of vascular endothelial cells, which play a vital role in building a functionally competent vascular network (49, 50). In this study, we demonstrated that laquinimod inhibited proliferation, migration and tube formation by HUVECs *in vitro* (Figures 5, 6). These data suggest that laquinimod also regulates the biological activity of vascular endothelial cells to inhibit CNV.

In conclusion, we found that laquinimod attenuated alkali-induced CNV and inhibited infiltrating inflammatory cells, such as macrophages, *in vivo*. Laquinimod also suppressed the expression of chemokines, inflammasomes and pro-inflammatory factors in injured corneas and RAW cells. Furthermore, laquinimod inhibited HUVEC proliferation, migration and tube formation *in vitro*. These data suggest that laquinimod is a potential new therapeutic option for CNV and other angiogenesis-associated diseases.

## Data Availability Statement

The raw data supporting the conclusions of this article will be made available by the authors, without undue reservation.

## Ethics Statement

The animal study was reviewed and approved by the Animal Ethical Committee at Zhongshan Ophthalmic Center of Sun Yat-sen University. Written informed consent was obtained from the owners for the participation of their animals in this study.

## Author Contributions

WS, YZhuo, and ZL contributed to the concept and/or design of the study. ZL, JC, LL, NJ, YZhu, and YJ contributed to the acquisition of the data, analysis, and interpretation. ZL, JC, and LL drafted the manuscript. YZhuo and WS reviewed and revised the manuscript. All authors read and approved the final version prior to submission.

## Conflict of Interest

The authors declare that the research was conducted in the absence of any commercial or financial relationships that could be construed as a potential conflict of interest.
